# The Association Between Nutritional Status, Diet Quality, and Sleep Quality Among the Elderly in Jordan: A Cross-Sectional Study

**DOI:** 10.1155/jare/7358242

**Published:** 2025-05-11

**Authors:** Shadan Al-Tal, Buthaina Alkhatib, Lana M. Agraib

**Affiliations:** ^1^Department of Clinical Nutrition and Dietetics, Faculty of Applied Medical Sciences, The Hashemite University, Zarqa, Jordan; ^2^Department of Nutrition and Food Science, Faculty of Allied Medical Sciences, Al-Balqa Applied University, Al-Salt, Jordan

**Keywords:** malnutrition, MEDAS, Mediterranean diet, MNA-SF, older adults, sleep quality

## Abstract

**Background:** The percentage of the elderly population has increased worldwide. It has been estimated that environmental factors such as eating habits, sleep quality, and physical activity could be responsible for up to 75% of the aging process.

**Aims:** To assess the nutritional status, diet quality, and sleep quality among the elderly in Jordanian.

**Methods:** In a cross-sectional study targeting the Jordanian elderly, 426 participants agreed to participate. Sociodemographic data, anthropometric measures, mini nutritional assessment—short form (MNA-SF), the Pittsburgh sleep quality index (PSQI), and the Mediterranean diet adherence screener (MEDAS) were measured.

**Results:** The mean and standard deviation were 10.7 ± 2.7 for the total MNA-SF score, 5.4 ± 1.8 for MEDAS, and 8.9 ± 4.2 for the global PSQI score. Most participants had normal nutrition status (43.9%) or were at risk of malnutrition (43.2%), 75.8% had poor sleeping quality, and 52.6% had poor MEDAS. There was a significant positive weak correlation between MNA-SF and MEDAS (*r* = 0.100, *p*=0.038) as well as the global PSQI score (*r* = 0.285, *p* < 0.001). On the other hand, there was a significant weak inverse association between MNA-SF and BMI (*r* = − 0.196, *p* < 0.001).

**Conclusion:** The Jordanian elderly had poor MEDAS adherence and poor sleeping quality, and this was correlated to an increased risk of malnutrition.

## 1. Introduction

With modern economic and medical advancements, human life expectancy has risen. Financial and healthcare progress has led to the swift growth of aging populations worldwide [1]. According to the World Health Organization (WHO), the percentage of the elderly population has increased worldwide; it is expected to rise from approximately 524 million in 2010 to almost 1500 million by 2050 [2]. According to Jordan's Department of Statistics (DOS), the elderly comprise about 3.7% of the Jordanian population. The elderly (65 years and older) increased from 206,510 in 2004 to 407,950 in 2021 [3]. In Jordan, the population has been proliferating due to the low mortality rate, high total fertility rate, and high rate of forced migration from surrounding countries, resulting in a relative increase in the proportion of elderly compared with other age groups [4].

Aging is usually associated with a progressive decline in the body's physiological, biological, and functional capacities, increasing the risk factors for various diseases [5]. The geriatric syndrome is among the most important problems affecting the older population and is linked to a reduction in life expectancy, difficulties carrying out routine tasks, and, in extreme situations, fatality. Dementia, starvation, insomnia, delirium, falls, syncope, dizziness, exhaustion, and incontinence are among its symptoms [6, 7].

It has been estimated that environmental factors such as eating habits, sleep quality, and physical activity may impact the normal aging process [8]. The impact of nutrition on physical and psychological well-being is most significant in the elderly and significantly contributes to health and functional ability [7].

The risk of malnutrition, which can be used to define an overall state of poor nutritional status involving both undernutrition and overnutrition of macro- and/or micronutrients [9], increases after 65 years of age, which increases an individual's risk of developing chronic diseases including sarcopenia and cardiovascular disease (CVD) [10]. The increasing prevalence of malnutrition among the elderly has been linked to several health problems, such as nutrient deficiency symptoms, loss of bone mass causing osteoporosis, immunological dysfunction, and slowed healing and recovery [11].

The term “diet quality” has become popular during the past 2 decades in the scientific literature; researchers have envisioned diet quality as a tool for assessing risk in predicting outcomes, including all-cause mortality, CVD, and cancer risk [12]. Also, sleep quality is another risk factor for malnutrition [13]. Sleeping disorders have become more prevalent and are among the most common health issues among the elderly [1]. Insufficient sleep duration was associated with diet and eating habits, causing individuals to consume more energy-dense meals or refined carbs and fewer fruits and vegetables [5, 14]. Studies on the association between sleep quality, diet quality, and nutritional status among the elderly are relatively rare. Due to this, the present study focused on this age group as it is viewed to be a neglected target category in the national dietary intervention programs [2]. As worldwide nutritional status and other environmental factors negatively enhance the normal age process, it is important to highlight the Jordanian elderly situation. This study aimed to evaluate the nutritional status, diet quality, and sleep quality of elderly individuals in Jordan and explore the relationships between diet quality, sleep quality, and nutritional status in this population.

## 2. Materials and Methodology

### 2.1. Study Design

A cross-sectional study that targeted the Jordanian elderly from different governorates was conducted between March and October 2023. The study population was all older adults randomly selected in Jordan, males and females aged 65 years and over [15].

Elderly participants or their relatives were interviewed and asked a list of questions. Before starting the questionnaire, each participant was provided with an overview of the study's objectives and how the study would add to the previous research, and they were asked to sign a written consent form. Their information remained anonymous. The Helsinki Declaration carried out all procedures, and the Institutional Review Board (IRB) by the Hashemite University Ethics Committee authorized the protocols, tools, and procedures for obtaining informed consent for this project (No. 10/1/2022/2023).

### 2.2. Study Sample

Due to the large size of the population under study and the difficulties in reaching the whole population, sampling was done. The sample size was calculated considering a confidence interval of 95% and a confidence level of 5% using Raosoft software (Raosoft, Inc. free online software, Seattle, WA, USA). The sample size was calculated according to Charan and Biswas [16], and the final sample size was 426.

### 2.3. Inclusion and Exclusion Criteria

Inclusion criteria were males and females aged 65 years old or above, living in Jordan, not hospitalized, and without severe dementia. The excluded participants were individuals with severe cognitive impairment, unable to interview the participant or his family, and conditions preventing anthropometric measurement, such as immobile patients.

### 2.4. Data Collection

The data collection was completed by face-to-face interviews assessing: (a) sociodemographic data; (b) anthropometric measures; (c) nutritional status assessment; (d) sleep quality; and (e) diet quality. The places for interviews included Hospitals such as Prince Faisal Hospital and Jabal Al-Zaytoon Hospital, in addition to health centers. In some cases, the meeting took place at the participant's homes. The interviews took place in a private, quiet, and appropriately sized room, free from distractions and located conveniently for all participants. They were conducted at a suitable time to minimize potential distractions during the data collection process [17].

Each interview lasted 15–25 min and was divided into two stages. The first involved anthropometric measurements (weight (kg), height (cm), body mass index (BMI), and calf circumference (CC) (cm)), and the second included gathering general data and the needed questionnaires. All data and information were double-checked before the interview ended.

In case of mild cognitive decline (which is defined as the in-between stage between typical thinking skills and dementia [18]) or if there was any problem understanding or answering specific questions, usually, a family member living with the participant in the same household was asked to answer these questions or even answer the questionnaire on behalf of the participant if this family member was fully responsible and took full care of the participant (*n* = 14 participants). The data collection procedure continued for approximately 7 months (March–October/2023).

### 2.5. Sociodemographic Data

Through the interviews, demographic data, including name, age, sex, marital status, educational level, employment status, lifestyle data (such as smoking status and physical activity), and medication information, were gathered.

### 2.6. Anthropometric Measurements and Indices

Height was measured by measuring tape, and the participants were standing with their backs against the wall while wearing light clothing and bare feet. The participants' legs were positioned so their knees and ankles touched each other. The height was measured and recorded to the nearest 0.1 cm. The study participants were weighed using a digital scale in the morning before eating and after going to the bathroom. Pieces of clothing that might affect the correct weight, such as shoes or heavy belts, were removed. The scale was tested and validated before the actual weighing of the study participant. The scale was set on a hard, flat surface. Each study participant was measured twice to confirm the accuracy of the recorded weight and recording to the nearest 0.1 kg.

BMI is the most frequently used method to diagnose malnutrition in the elderly and indicates appropriate weight for height. BMI was calculated by dividing the individual's weight by the square of their height (kg)/(m^2^) and categorized as underweight (BMI < 18.5 kg/m^2^), normal weight (BMI 18.5–24.9 kg/m^2^), overweight (BMI 25–29.9 kg/m^2^), and obese (BMI ≥ 30 kg/m^2^ and more) [19, 20]. BMI was also used in the mini nutritional assessment—short form (MNA-SF) (MNA-SF); points were scored as follows: 0 = BMI less than 19; 1 = BMI 19 to less than 21; 2 = BMI 21 to less than 23; and 3 = BMI 23 or greater. If BMI was not available, CC was measured.

However, CC is a specific marker for sarcopenia and has demonstrated a strong association with serum albumin and BMI [21]. In such cases, when we faced difficulty measuring some participants' accurate and correct weight, we resorted to calculating the CC. However, the BMI was adopted primarily. For MNA-SF, points were scored as follows: 0 = CC less than 31 and 3 = CC 31 or greater (*n* = 5 participants).

### 2.7. Nutritional Status Assessment

To assess the nutritional status and the risk of malnutrition in the participants, the Arabic version of the MNA-SF tool was used as a valid method to evaluate the nutritional status of the elderly [22]. The MNA-SF is a simple, inexpensive, and noninvasive screening tool for assessing malnutrition and the risk of malnutrition in older people [5]. It consists of six items: mobility limitation, acute illness/stress, dementia or depression, appetite or eating issues, and weight loss in the previous 3 months, and BMI or CC [1]. All items were administered to elderly individuals. All MNA-SF items have been derived from the full MNA-SF [23]. All anthropometric measurements involved in MNA-SF were performed at the beginning of the interview. MNA-SF has a maximum of 14 points; the scores were calculated as the sum of points assigned to the responses of 6 items. According to the nutritional status, this assessment is classified into three categories: normal (12–14 points), at risk of malnutrition (8–11 points), and malnutrition (0–7 points) [21, 24].

### 2.8. Sleep Quality Assessment

The validated Pittsburgh sleep quality index (PSQI) questionnaire assessed sleep quality and identified sleep disorders. The PSQI consists of seven component scores, comprising 19 questions of self-evaluation involving subjective sleep quality, sleep duration in hours during the night, sleep efficiency, sleep disturbances (for several causes), sleep latency, daytime dysfunction, and use of sleep medications (prescribed by a physician) [1]. A PSQI score of 5 or less reflects good sleep quality, while a score above 5 indicates poor sleep quality [5].

### 2.9. Diet Quality Assessment

The Mediterranean diet score assessed the diet quality. The validated Arabic version of the Mediterranean diet adherence screener (MEDAS) was used. MEDAS includes several food groups, such as olive oil, fruits, vegetables, legumes, fish, meat, and tomato-based sauce, featured in MEDAS [25]. A pilot study with a subset of the intended population of almost 31 participants was completed to validate the questionnaire. Initially, the questionnaire was translated by experts from its validated version in English into Arabic; the translated version was reviewed before being approved and used; then it was translated back to English after completing data collection and before the analysis. Thirty-one participants, including 17 women and 14 men, all over 65 years old, filled out the questionnaire; after that, we ran the statistics on their responses and entered the data. Three weeks after filling out the questionnaire, we contacted them to repeat the questionnaire under the same conditions to see if we had the same results as the first time. Using Cronbach's alpha test, the alpha value was 0.76, indicating acceptable reliability [26]. There are 12 items related to food consumption frequency and two eating behaviors with a score of 0 or 1; for each answer that matches the MEDAS, one point is given, resulting in scores ranging from 0 to 14. For example, one point was given out for preferring white meat over red meat and another for using olive oil as the primary cooking fat source. The following criteria were used to classify adherence to the MEDAS: poor adherence ≤ 5, moderate to fair adherence 6–9, and good or very good adherence ≥ 10 [27].

### 2.10. Statistical Analysis

Data were analyzed using SPSS software (IBM SPSS Statistics for Windows, Version 25.0. Armonk, NY: IBM Corp.). The chi-square test (*χ*^2^) or Fisher exact test was used to assess differences between categorical variables, and the results were presented as percentages. In contrast, continuous variables were analyzed using an independent *t*-test or one-way analysis of variance (ANOVA) and were described using means ± standard deviation (SD). Correlations were evaluated using Spearman's correlation coefficient. A *p* value < 0.05 was considered statistically significant.

## 3. Results

Of the total 426 older adults who took part in the study, the mean weight was 79.7 ± 18.3 kg, the height was 163.9 ± 9.2 cm, and the BMI was 29.8 ± 6.8 kg/m^2^, and there were 122 (28.6%) male and 304 (71.4%) female (Table 1). About half of the study participants (49.5%) were 65–75, 34.3% were aged 71%–80%, and 16.2% were aged above 81 years. Based on BMI classification, 36.2% of the participants were overweight, 41.8% were obese, and 22.1% were of normal weight. The educational level of the participants was 23.2% illiterate, 33.1% either can read and write or primary, 20.9% secondary, and 22.8% have university education. Most participants were married (49.3%) or widowed (47.7%). Seventy-eight percent of the participants lived in central cities and were not smokers. Regarding work, most of them do not have a job (64.1%) or have retired (30.8%). In addition, 75.8% of participants had governmental health insurance. Additionally, the mean scores were 10.7 ± 2.7 for MNA-SF, which indicates a normal to at-risk nutritional status, 5.4 ± 1.8 for MEDAS, which indicates poor to moderate diet quality, and 8.9 ± 4.2 for the global PSQI score, which indicates poor sleep quality (Table 1).

**Figure 1:**
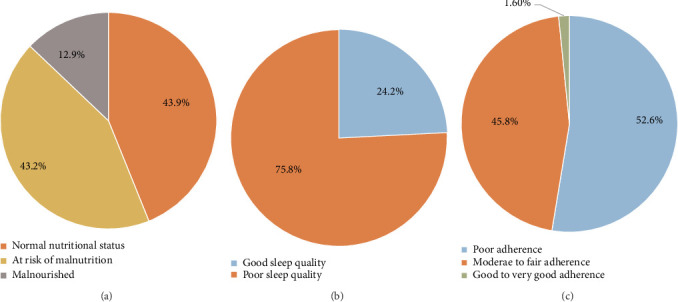
The prevalence of (a) nutritional status assessment (MNA-SF); (b) Pittsburgh sleep quality index (PSQI); and (c) the Mediterranean diet adherence screener (MEDAS).

The mean and SD for the total MNA-SF score were 10.7 ± 2.7, 5.4 ± 1.8 for MEDAS, and 8.9 ± 4.2 for the global PSQI score. As shown in Figure 1, most participants either had normal nutrition status (187 (43.9%)) or were at risk of malnutrition (184 (43.2%)), and only 55 (12.9%) of them were malnourished (Figure 1(a)). Regarding the global PSQI score, 323 (75.8%) of the participants had poor sleeping quality, and just 103 (24.2%) had good sleeping quality (Figure 1(b)). Based on adherence to the Mediterranean diet, 224 (52.6%) of participants had poor adherence, 195 (45.8%) had moderate to fair adherence, and only 7 (1.6%) had good to very good adherence (Figure 1(c)).

The Spearman rho correlation test between MNA-SF, MEDAS, and global PSQI is shown in Table 2. There was a significant positive weak correlation between MNA-SF and MEDAS (*r* = 0.100, *p*=0.038) as well as the global PSQI score (*r* = 0.285, *p* < 0.001). On the other hand, there was a significant weak inverse association between MNA-SF and BMI (*r* = − 0.196, *p* < 0.001). Additionally, no other correlations were significant, apart from those mentioned above.

Using MNA-SF as a dependent (Table 3), the prevalence of moderate to fair, and poor adherence was high in all groups of MNA-SF. However, participants with normal nutritional status had a higher prevalence of moderate to fair adherence (51.3%), while malnourished participants had a higher prevalence of poor adherence (61.8%), but this was not significant (*p*=0.14). Participants with normal nutrition status had significantly higher prevalences of good sleeping quality (37.4%) compared to malnourished participants (5.5%). In comparison, the last one had significantly higher prevalences of poor sleeping quality (94.5% vs. 62.6% for participants with normal nutrition status) (*p* < 0.001).

## 4. Discussion

The body's physiological, biochemical, and functional capacities often deteriorate with age, raising the risk factors for several disorders [5]. According to estimates, it has been approved that environmental factors, including sleep patterns, physical activity levels, and eating habits, may have an important impact on the normal aging process [8]. Nutrition significantly influences health and functional ability and has a greater effect on psychological and physical well-being in the elderly [7]. Elderly malnutrition is becoming more common, and this has been connected to many health issues, including immunological dysfunction, osteoporosis, signs of vitamin deficiencies, and slower healing and recovery [11]. Therefore, the present study aimed to assess the nutritional status, diet quality, and sleep quality among Jordanian elderly people.

The present findings indicated that most participants had normal nutrition status or were at risk of malnutrition. As current findings, the prevalence of malnutrition in the Jordanian elderly (12.9%) was worse than that of the elderly in most countries, such as Brazil (3.1%) [28], Turkey (8.4%) [29], Mexico (4.9%) [30], Saudi Arabia (9.6%) [31], and Taiwan (0.7%) [32]. The prevalence of malnutrition among the present study participants was better than in Iraq (24.0%) and China (52.7%) [33]. In comparison, the prevalence of at-risk malnutrition (43.2%) in the present findings is considered higher than the findings of Brazil (35.4%) [28], Turkey (37.0% and 34.2%) [29, 34], China (27.5%) [33], Croatia (30%) [35], Mexico (31%) [30], Saudi Arabia (27.5%) [31], and Vietnam (21%) [36, 37]. However, the nutritional status was negatively correlated to BMI (the majority of participants were overweight and obese) in the current study. This finding was in line with Bahat and colleagues' findings, who reported that relatively high rates of undernutrition could exist in overweight or obese BMIs in the Turkish elderly [38]. Also, de Morais and colleagues found that the lowest nutritional risk was shown in BMI around 18.5 kg/m^2^ [32]. In Poland, it has been found that BMI values were significantly correlated to MNA-SF scores [39]. It has been found that 34.8% of those with a malnourished MNA score had higher BMI values [40]. However, Alemán-Mateo and colleagues found that the elderly at risk of undernutrition had significantly lower body weight, BMI, total body fat, and waist circumference [30].

Regarding adherence to the Mediterranean diet, about half of the participants in the present study had poor adherence. Only a tiny percentage of participants have good adherence to MEDAS (1.6%). Also, it was positively correlated to nutritional status. The prevalence of poor adherence to MEDAS was higher in malnourished participants. The present findings were not surprising, as the dietary patterns and eating habits among the elderly have shifted worldwide. Ergul and colleagues (2022) approved that the rate of unhealthy diets among the Turkish elderly was 91.5%, and 17% were malnourished. Eating dietary index was significantly and positively correlated with the MNA-SF score [41]. It has been found that poor Mediterranean diet adherence is linked to adverse changes in body composition and circulating pro-inflammatory markers in older Italian patients admitted to internal medicine wards and more extended hospital stays [42]. According to the MNA scale, 19% of the elderly in Spain were malnourished, and 68% showed signs of potential malnutrition. Of the 111 participants, 27% demonstrated moderate adherence to the Mediterranean diet, and 73% showed low adherence [43]. Moreover, the elderly population of Villanueva is moving away from the traditional Mediterranean diet. While adequate consumption of fruits, dairy products, oils/fats, and sugar/confectionery has been achieved, the consumption of cereals/grain products, vegetables, and the meat, fish, and eggs group was below the desirable levels. A negative association between milk/dairy products and MNA was also found in this population. Fruit consumption significantly decreased as nutritional status worsened (*p*=0.039). MNA was found to have fragile but significant relationships with oils/fats, fruits, and alcoholic beverages [44]. On the other hand, Madiera and colleagues assessed the dietary patterns among the elderly in Portugal, and two dietary patterns were identified: 22.0% of the studied population followed a protein-based foods dietary pattern, and 59.1% followed a Mediterranean dietary pattern [45]. Moreover, 18.9% switched between two patterns. The protein-based foods dietary pattern was associated with a better MNA score and lower malnutrition risk, compared to the Mediterranean dietary pattern, particularly for total energy intake up to 2200 kcal/day [45].

However, the present findings demonstrate a nonsignificant correlation between MEDAS and BMI. It has been found that adherence to the Mediterranean diet is associated with an 88% (*p*=0.07) lower likelihood of being obese among elderly people living on Mediterranean islands [46]. Moreover, in Italian older adults, a high adiposity was found in 971 (46.4%) participants and was more frequent in those with a low (54.2%) or moderate (46.4%) Medi-Diet adherence compared with the high-adherence group (39.7%, *p*  <  0.001). Logistic regression indicated that older adults with high Medi-Diet adherence were less likely to have a high relative fat mass [47].

The present findings indicated that most elderly participants had poor sleeping quality (75.5%). Of the elderly Korean population, approximately 12.5%, 22%, and 51.3% had poor sleep quality, excessive daytime sleepiness, and insomnia; respectively [48]. The prevalence of poor sleep quality varied between countries: 42.6% in Iran [49], 61.6% of Turkish elderly [50], and 31.5% in China [51]. In the present correlations, poor sleep quality was positively correlated to nutritional status. Moreover, normal nutritional status had significantly higher prevalences of good sleeping quality compared to malnourished participants, and the opposite is true. This study's findings were consistent with those from other studies conducted in the same field worldwide. In the Turkish elderly, the MNA score was lower among the poor sleep quality group, and the PSQI score negatively correlated with MNA [52]. In Greece's elderly, a better nutritional status was significantly and independently associated with better sleep quality (*p*=0.0202) [50]. They approved a strong correlation between sleep quality and nutritional status in China; the findings indicated that maintaining a healthy diet was linked to lower development of sleep disorders. A healthy nutritional status was substantially related to reduced dysfunction during the day, sufficient sleep duration, and favorable subjective sleep quality (all *p*  <  0.05) [53]. Additionally, multiple logistic regression analysis demonstrated that participants who were malnourished had poor sleep quality among older adults in Thailand [51]. The current findings indicate a nonsignificant correlation between sleep quality and BMI, consistent with Li and colleagues' findings among Chinese elderly in rural areas [54]. On the other hand, Taheri and Irandoust found that the exercise-induced weight loss improved sleep quality in obese elderly women [55]. Additionally, Liang and colleagues concluded that overweight or obese Chinese elderly may have better sleep quality compared to their normal weight counterparts, whereas underweight elderly individuals may experience poorer sleep quality [56]. In the elderly Iranian population, it has been found that the effect of BMI on sleep quality is sex-based; men showed positive associations between the third tertile of poor sleep quality and BMI. Still, they did not demonstrate an association between sleep quality and body composition components. Meanwhile, in women, there were positive associations for BMI and negative associations for skeletal muscle [57].

### 4.1. Strengths and Limitations

The current study has certain limitations; because older people's height decreases with age, it might be challenging to determine their actual height from a standing position. In addition, despite being collected, information on the medication was not used during data analysis. The study's drawback was its inability to reach single individuals, who are unquestionably more vulnerable to malnutrition than those who live with their families; establishing a cause-and-effect link was impossible because this study was cross-sectional; gaining the findings to other regions will be challenging because the survey was conducted in a narrow geographic area; and some diseases may independently affect sleep quality and could act as confounding variables in this study, which were not mentioned research. Moreover, no data for the hip–waist ratio was reported due to incomplete measurements of all the participants and some questionnaires being filled out by a family member. The current study also has some ramifications. The results of this study will assist policymakers in developing plans for the inclusive involvement of primary care physicians in the early detection and management of cases of malnutrition among the elderly. However, this was the first study that correlated sleep quality, nutritional status, and adherence to the Mediterranean diet among Jordanian elderly.

## 5. Conclusions

Although the prevalence of malnutrition among the elderly is lower (12.91%), about 40% were at risk of malnutrition. Malnutrition was positively correlated with poor sleep quality and poor adherence to the Mediterranean diet and negatively correlated with BMI. Future studies are needed for the elderly, considering biochemical data, existing chronic diseases, medication use, wide geographic coverage, and sex-based analysis.

## Figures and Tables

**Table 1:** The general characteristics of the sample population (*N* = 426).

**Variables**	**Mean ± SD**

Weight (kg)	79.7 ± 18.3
Height (cm)	163.9 ± 9.2
Body mass index (kg/m^2^)	29.8 ± 6.8
MNA-SF	10.73 ± 2.7
MEDAS	5.4 ± 1.8
PSQI	8.9 ± 4.2

**Variables**	***n* (%)**

*Sex*	
Males	122 (28.6)
Females	304 (71.4)

*Age group*	
65–70 years	211 (49.5)
71–80 years	146 (34.3)
81 years and above	69 (16.2)

*Body mass index categories*	
Normal	94 (22.1)
Overweight	154 (36.2)
Obese	178 (41.8)

*Educational level*	
Illiterate	99 (23.2)
Can read and write	60 (14.1)
Primary school	81 (19.0)
Secondary school	89 (20.9)
University (bachelor's and higher education)	97 (22.8)

*Marital status*	
Married	210 (49.3)
Single	4 (0.9)
Divorce	9 (2.1)
Widow	203 (47.7)

*Place of residence*	
Central	333 (78.2)
Northern	85 (20.0)
Southern	8 (1.9)

*A smoker*	
Yes	93 (21.8)
No	333 (78.2)

*Work status*	
Governmental	5 (1.2)
Private	17 (4.0)
Retired	131 (30.8)
Not working	273 (64.1)

*Do you have health insurance?*	
Yes, governmental	323 (75.8)
Yes, private	42 (9.9)
No	61 (14.3)

Abbreviations: MEDAS, the Mediterranean diet adherence screener; MNA-SF, mini nutritional assessment; PSQI, Pittsburgh sleep quality index; SD, standard deviation.

**Table 2:** The Spearman rho correlation test between MNA-SF, MEDAS, and global PSQI.

		MNA-SF	MEDAS	Global PSQI score	BMI
MNA-SF	*r*		0.100^∗^	0.285^∗∗^	−0.169^∗∗^
	*p* value		0.038	< 0.001	< 0.001

MEDAS	*r*	0.100^∗^		−0.013	−0.076
	*p* value	0.038		0.796	0.118

Global PSQI score	*r*	0.285^∗∗^	−0.013		0.022
	*p* value	< 0.001	0.796		0.655

BMI	*r*	−0.169^∗∗^	−0.076	0.022	
	*p* value	< 0.001	0.118	0.655	

Abbreviations: BMI, body mass index; MEDAS, the Mediterranean diet adherence screener; MNA-SF, mini nutritional assessment; PSQI, Pittsburgh sleep quality index.

^∗^Correlation is significant at the 0.05 level (2-tailed).

^∗∗^Correlation is significant at the 0.01 level (2-tailed).

**Table 3:** The prevalences of MEDAS and global PSQI score categories among MNA-SF groups.

	MNA-SF			*p* value^∗^
	Normal nutritional status	At risk of malnutrition	Malnourished	
*MEDAS categories*				
Good to very good adherence	3 (1.6)	2 (1.1)	2 (3.6)	0.14
Moderate to fair adherence	96 (51.3)	80 (43.5)	19 (34.5)	
Poor adherence	88 (47.1)	102 (55.4)	34 (61.8)	

*Global PSQI score categories*				
Good sleeping quality	70 (37.4)	30 (16.3)	3 (5.5)	< 0.001^∗^
Poor sleeping quality	117 (62.6)	154 (83.7)	52 (94.5)	

*Note:* Data are presented as *n* (%).

Abbreviations: MEDAS, the Mediterranean diet adherence screener; MNA-SF, mini nutritional assessment; PSQI, Pittsburgh sleep quality index.

^∗^The value is considered significant at *p* < 0.05.

## Data Availability

The data presented in this study are available on request from the corresponding author.

## Nomenclature

BMI: Body mass index
CC: Calf circumference
CVD: Cardiovascular disease
MEDAS: Mediterranean diet adherence screener
MNA-SF: Mini nutritional assessment—short form
PSQI: Pittsburgh sleep quality index
